# Fetal Cardiac Services during the COVID-19 Pandemic: How Does It Affect Parental Counseling?

**DOI:** 10.3390/jcm10153423

**Published:** 2021-07-31

**Authors:** Alexander Kovacevic, Stefan Bär, Sebastian Starystach, Michael Elsässer, Thomas van der Locht, Aida Mohammadi Motlagh, Eva Ostermayer, Renate Oberhoffer-Fritz, Peter Ewert, Matthias Gorenflo, Annette Wacker-Gussmann

**Affiliations:** 1 Department of Pediatric and Congenital Cardiology, Heidelberg University Hospital, 69120 Heidelberg, Germany; thomasvanderlocht@yahoo.de (T.v.d.L.); Matthias.Gorenflo@med.uni-heidelberg.de (M.G.); 2 Max Weber Institute for Sociology, Ruprecht Karls University Heidelberg, 69115 Heidelberg, Germany; stefan.baer@mwi.uni-heidelberg.de (S.B.); sebastian.starystach@charite.de (S.S.); 3 Institute of Medical Sociology and Rehabilitation Science, Charité—University Medicine Berlin, 10117 Berlin, Germany; 4 Department of Gynecology and Obstetrics, Heidelberg University Hospital, 69120 Heidelberg, Germany; Michael.Elsaesser@med.uni-heidelberg.de; 5 Institute of Preventive Pediatrics, Faculty of Sport and Health Sciences, Technical University of Munich, 80992 Munich, Germany; aida_motlagh@yahoo.de (A.M.M.); renate.oberhoffer@tum.de (R.O.-F.); annette.wacker-gussmann@tum.de (A.W.-G.); 6 Department of Pediatric Cardiology and Congenital Heart Defects, German Heart Center Munich, 80636 Munich, Germany; ewert@dhm.mhn.de; 7 Department of Obstetrics and Gynecology, Klinikum Rechts der Isar, Technical University of Munich, 81675 Munich, Germany; eva.ostermayer@t-online.de

**Keywords:** COVID-19 pandemic, fetal congenital heart disease, prenatal diagnosis, parental counseling

## Abstract

The COVID-19 pandemic impacts health care providers in multiple ways, even specialties that do not seem to be affected primarily, such as fetal cardiac services. We aimed to assess the effects on parental counseling for fetal congenital heart disease (CHD). In this multicenter study, we used a validated questionnaire. Parents were recruited from four national tertiary medical care centers (*n* = 226); *n* = 169 had been counseled before and *n* = 57 during the pandemic. Overall counseling success including its dimensions did not differ between the two groups (*p* = n.s.). However, by applying the sorrow scale, we could demonstrate that parents counseled during the pandemic were significantly more concerned (*p* = 0.025) and unsure (*p* = 0.044) about their child’s diagnosis, therapy and outcome. Furthermore, parents expressed a significantly increased need for written and/or online information on fetal heart disease (*p* = 0.034). Other modifiers did not affect counseling success (*p* = n.s.). We demonstrate that the COVID-19 pandemic impacts effectiveness of parental counseling for fetal CHD, possibly by altering parental perceptions. This needs to be taken into consideration when counseling. Implementing alternative and innovative approaches (e.g., online conference or virtual reality tools) may aid in facilitating high-quality services in critical times such as in the present pandemic.

## 1. Introduction

Congenital heart defects (CHD) are the most common form of congenital anomalies and can be routinely diagnosed during prenatal screening [1,2]. Hereafter, effective parental counseling forms a crucial part in fetal cardiac services [3,4,5].

The COVID-19 pandemic impacts national health care systems in multiple ways, and fetal cardiac services do not seem to be affected primarily.

Effective parental counseling is considered essential and has the potential to impact pregnancy and postnatal outcomes. In a subset of mothers, experiencing a diagnosis of CHD in their unborn child leads to increased stress, anxiety and depression [6,7,8,9,10,11,12].

Successful parental counseling is dependent on variable factors and has been inconsistently realized, even before the pandemic. International guidelines aid in improving the counseling process, and modifiers affecting effectiveness—such as the availability of a separate counseling room, adequate length of counseling sessions, the severity of diagnosed CHD or the consultants’ individual skills—have been identified [4,5,13,14,15,16].

The COVID-19 pandemic has great potential to further raise concerns among pregnant women and their partners while expecting a newborn with CHD. Prophylactic measures applied during this pandemic, such as physical distancing, home isolation, quarantine, or complete lockdown periods are additional risk factors that may affect mental health. Recommendations for the management of pregnancy during COVID-19 vary due to a lack of robust evidence, and these uncertainties are likely to additionally increase parental anxiety and stress [17]. Peak anxiety scores among pregnant women are observed, e.g., during lockdowns or periods with higher numbers of COVID deaths reported [18]. However, being pregnant does not seem to increase the risk of a coronavirus infection per se, but it has been shown that pregnant women with COVID-19 were more likely to be hospitalized and require intensive care unit (ICU) admission than non-pregnant women. A mild increase in preterm deliveries has been observed, but not for pregnancy loss rates [19,20,21]. Further, the risk of vertical transmission seems low [22]. However, current upcoming SARS-CoV-2 (Severe Acute Respiratory Syndrome Coronavirus-2) variants of concern with reported increased transmission rates are potentially causing even more uncertainty, while on the other hand, COVID-19 vaccination strategies for pregnant women differ between national health care systems.

Still, despite the increasing number of studies, data seem insufficient to draw general conclusions with regard to complications of COVID-19 in pregnant women [23]. Moreover, assessments of the efficacy of parental counseling strategies for fetal CHD during the pandemic are lacking, but worthy of further exploration.

Therefore, the purposes of this study were first to assess the success of parental counseling for fetal heart disease during the pandemic and to compare results with data acquired before the pandemic. Second, we hoped to identify emerging parental needs in this context with the aim to adapt the counseling process consequently.

We hypothesized that the present COVID-19 pandemic impacts the efficacy of parental counseling for fetal CHD.

## 2. Materials and Methods

We prospectively recruited parents from four national tertiary medical care centers (Figure S1: study flow-chart). Parental counseling for fetal CHD was assessed by interviewing parents during routine follow-up visits of their children to Pediatric Heart Centers (by two co-authors who were not involved in fetal care to avoid response bias). Alternatively, questionnaires were issued during the visits or were sent to the families. Parents from both subgroups (group one: parents counseled before COVID-19; group two: parents counseled during COVID-19) were counseled by the same three experienced fetal/pediatric cardiologists and three maternal-fetal medicine (MFM) specialists, either separately or together. Inclusion criteria were parental age of at least 18 years and custody of the child, cardiac malformations detected in prenatal screening, parental counseling between 2009 and 2019 during the “pre-COVID era” and 1 March 2020—1 December 2020 for the COVID subgroup. We excluded cases with severe extracardiac malformations or genetic syndromes (such as Trisomy 13 or 18) with an unfavorable outcome as sole diagnosis, thus affecting overall postnatal outcome and therefore counseling.

Counseling success was measured using sum scores from items taken from the Likert scale questionnaire validated previously, which was developed by a multidisciplinary team of sociologists, pediatric and fetal cardiologists, as well as MFM specialists (Figure S2: design of the questionnaire). By Likert-scaling, the extent of parental agreement or disagreement with each question or statement can be measured on a five-point scale: strongly agree, agree, partially agree, disagree, and strongly disagree. For statistical analysis, each point scale is converted into a number from one to five. Items were constructed as indicators showing success when answered as positive in the two assenting values of the Likert response format. The variable “overall counseling success“ was constructed by building a sum score of the 16 items from the questionnaire by transforming it into an ordinal scaled variable defining the respective range 16–32 as “successful”, 33–63 as “satisfying” and 64–80 as “unsuccessful”. In the same way, we constructed the variables for the five dimensions of counseling success, whereby the number of items varied [15].

The parental native language was documented, and the questionnaire ended with an open-ended question asking for feedback and additional remarks.

The questionnaire’s internal consistency was tested with this new dataset. Cronbach’s alpha (=reliability coefficient) was 0.904, thus displaying the scales’ high internal consistency [24].

The non-parametric Mann–Whitney U-Test was used to compare differences between two independent samples (no normal distribution of the dependent variable, which is ordinal scaled) by analyzing the mean ranks for the ordinal-scaled dependent variable (counseling success). Reported are the rank sums (which are in accordance with the coding of the dependent variable; lower values of mean ranks tend to indicate better counseling success) and the significance level. If *p* is less than 0.05, the hypothesis of difference between the two groups is confirmed. Statistical analyses were performed using IBM^®^ SPSS^®^ Statistics Version 25 (IBM Germany, Ehningen).

Participants gave their written informed consent before inclusion in the study, which was conducted in accordance with the Declaration of Helsinki. The study protocol was approved by the local Ethics Committee of each involved Medical Faculty (reference numbers: S-250/2016 and 341/18 S-KK).

## 3. Results

*n* = 226 parents were recruited, *n* = 169 had been counseled before and *n* = 57 during the pandemic. Fetal gestational age at the time of diagnosis of fetal CHD and parental counseling was 23 weeks (median) before and 22 weeks (median) during the pandemic. Details on fetal cardiac diagnoses, extracardiac anomalies and genetic syndromes are summarized in Table S1. Parents from both subgroups had been counseled by the same three consultants. The median parental age at the time of counseling for fetal heart disease was 35 years before and 34 years during the pandemic. Mothers had been counseled more often compared to fathers (before COVID-19: female 59.2% vs. male 40.8%; during COVID-19: female 59.6% vs. male 40.4%). The gender distribution was similar in both groups.

Overall counseling success did not differ significantly between these two groups, and there was no statistically significant difference for the analytical dimensions (Table 1).

However, applying the sorrow scale reveals that parents having their fetuses diagnosed with CHD during the pandemic were significantly more concerned (*p* = 0.025) and unsure (*p* = 0.044) about the child´s diagnosis, future therapy and outcome (Table 2), while the effect sizes (r calculated from the magnitude of Z divided by the square root of N) are relatively small (0.157 and 0.142, respectively). Parental perceptions of the seriousness of the situation did not differ significantly between the two groups (*p* = not significant, n.s.).

Counseling was not affected significantly during the pandemic by modifiers such as interruptions during the counseling, the availability of a separate counseling room or timely counseling by a specialist after suspicion of fetal CHD (Table 3; *p* = n.s.). However, there was a significantly increased parental demand for written or online information on the cardiac diagnosis, treatment and prognosis of the disease (Table 3; *p* = 0.034).

During the investigation period before the pandemic, 60.7% of fetuses were diagnosed with complex CHD, and 46.4% during the pandemic (according to Allan and Huggon [3]). Mean values for parental overall counseling success and the analytical dimensions were similar before and during the pandemic for both groups of parents, indicating no significant differences (Table 4).

**Figure 1:**
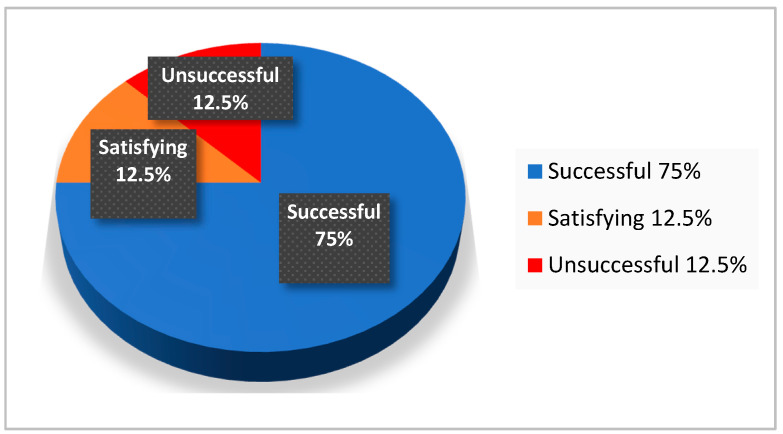
Distribution of counseling success (1. successful, 2. satisfying, 3. unsuccessful) for “parental perceived situational control” with regard to parental native language (i.e., parental native language differing from the language counseling was conducted in).

Parental native language was not identical with the language counseling was conducted in 14% of parents during the COVID-19 pandemic. Nonetheless, counseling success was high (successful and/or satisfying) for overall counseling success and in four analytical dimensions. Counseling success in terms of achieving a successful parental “perceived situational control” was unsuccessful in 12.5% (Table 5 and Figure 1; cases were only included if parental language skills appeared to be sufficient during counseling).

**Figure 2:**
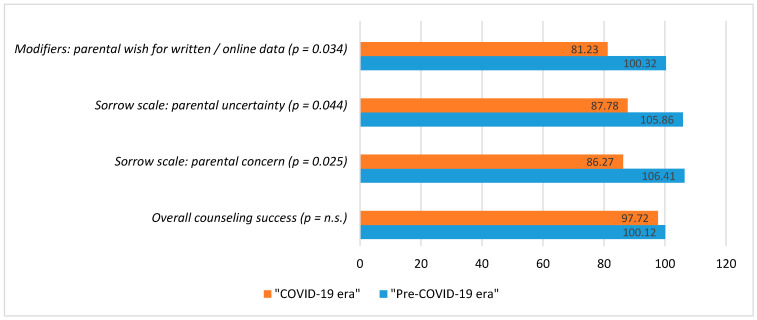
Overview of key results. Mean ranks displayed as column charts incl. *p*-values (Mann Whitney-U Test) for “Overall Counseling Success“, “Sorrow scales“ and selected modifiers for parental counseling success in the two subgroups.

Figure 2 summarizes some key results with mean ranks displayed as column charts.

## 4. Discussion

Having one’s own unborn child diagnosed with CHD is undoubtedly a traumatic event for parents, and potential effects on the mother’s, father’s, fetal and newborn’s health have been described [6,7,8,9,10,11,12]. Being pregnant during the COVID-19 pandemic brings additional factors that may affect in particular maternal mental health.

This moved us to assess parental counseling for fetal CHD in this context, with the aim to proactively adapt counseling strategies. 

There are several novel findings in this study concerning parental counseling for fetal heart disease during the COVID-19 pandemic.

First, the pandemic has impacted the counseling process. Parents were significantly more concerned and unsure about the cardiac diagnosis, future therapy and outcome of their unborn child, with an increased demand for explanatory written or online information (Figure 2).

Second, neither counseling success in general nor any of its dimensions were significantly affected during the pandemic.

Third, modifiers of counseling success such as interruptions during counseling, the availability of a separate counseling room or severity of diagnosed fetal CHD did not have any significant effects.

Fourth, the multicenter and particularly the interdisciplinary approach of this study has proven to be extremely useful in analyzing comprehensively central issues of parental counseling for fetal CHD in the context of a pandemic.

Additionally, this is the first study in which the potential effect of the COVID-19 pandemic on parental counseling in this setting has been analyzed.

Before the pandemic, our group was able to show that modifiers of counseling success—such as a lack of a separate counseling room or complexity of CHD—impacted effectiveness negatively [15]. This, however, could not be reproduced in the COVID-19 subgroup. The different influence of these moderators is likely to be explained by the fact that during the pandemic, day-to-day operations were reduced (i.e., elective routine check-ups have been postponed temporarily), therefore possibly leading to a more stable consulting setting. If this hypothesis holds true, returning to the routine after the pandemic will increase again the importance of these variables. Therefore, in the future, MFM-specialists and cardiologists should still make sure that counseling in the aforementioned cases of CHD should be carried out in a structured way. It further cannot be ruled out that consultants in general have adapted their counseling strategies and improved skills over time, or in view of the pandemic, have been particularly motivated.

On the other hand, parents counseled during the COVID-19 pandemic were significantly more concerned and unsure about their child´s diagnosis, future therapy and outcome. They expressed a significantly increased need for written or online information on the cardiac diagnosis, treatment and prognosis, compared to the pre-COVID-19 era. 

From our data we may conclude that the COVID-19 pandemic affects the counseling setting for fetal heart disease, primarily in a negative way (additional uncertainty and concern), but also potentially in a positive way (stability) for the above mentioned reasons, therefore leading to the outcome that neither overall counseling success nor any of its dimensions were significantly affected.

In summary we believe that fetal cardiologists and maternal–fetal medicine specialists need to appreciate the additional external circumstances of this pandemic. Explanatory material (written or online) is considered beneficial and has been proven useful in earlier studies [14,15]. As parental concern seemed higher during the pandemic, we believe that early psychological support is even more important than before. Therefore, the structured approach for parental counseling for fetal heart disease that was proposed by our group earlier appears to be even more important in the context of the pandemic [15]. 

Regulations by hospitals to avoid repetitive contacts to reduce potential infectious exposure for both pregnant mothers and physicians may point the way to an increased use of telephone or online conference tools, including innovative technologies such as virtual hospital visits with communication on portable devices. Some centers increasingly use telemedicine services, reportedly without compromising diagnostic accuracy. However, a strong infrastructure for implementation is required [25,26]. Once the pandemic has passed, usage of this technology may even remain attractive for both parents and medical staff.

### Limitations

Due to the sample size and sampling method, the generalizability of our findings may be diminished.

Skills regarding counseling may differ among physicians, and personal bias may have been imposed. However, in all centers included in this study, nondirective counseling is compulsory.

Adverse effects of the pandemic on mental health of health care providers with higher levels of anxiety and depression, as well as high work exhaustion, have been reported [27]. We did not include these as variables, but our results suggest no differences for overall counseling success, so it may be speculated that the effect on this dataset is minor.

Parental religious, cultural, ethnic or socio-economic backgrounds may have influenced parental judgment. Specific data for these factors are not included but will be analyzed in a forthcoming study.

## 5. Conclusions

Analyses of the impact of the COVID-19 pandemic on medical specialties, which do not seem to be affected primarily, seem worthwhile. For this purpose, our interdisciplinary approach has proven to be extremely useful. Fetal cardiac services are not only affected by the risk of a SARS-CoV-2 infection of the mother, the fetus or the newborn. Parental perceptions seem to be altered by the pandemic, and this needs to be taken into consideration when counseling for fetal heart disease. Adapting the framework, e.g., implementing alternative and innovative approaches such as online conference or virtual reality tools, will aid in facilitating high-quality services in critical times such as the ongoing COVID-19 pandemic.

## Figures and Tables

**Table 1:** Overall parental counseling success (a) and counseling success for the analytical dimensions (b) did not differ significantly between parents counseled before and during the COVID-19 pandemic.

Counseling Success			
	Before COVID-19	During COVID-19	
*n* = 198	147	51	
	Mean rank		*p* (Mann–Whitney-U)
(a) Overall counseling success	100.12	97.72	0.766
(b) Dimensions:			
Transfer of medical knowledge	100.58	96.38	0.605
Trust in medical staff	101.16	94.72	0.366
Transparency regarding the treatment process	99.32	100.01	0.930
Coping resources	99.99	98.10	0.818
Perceived situational control	100.29	97.23	0.721

**Table 2:** Sorrow scale testing and results in parents counseled before and during the pandemic.

Sorrows			
	Before COVID-19	During COVID-19	
*n* = 201	147	54	
	Mean rank		*p* (Mann–Whitney-U)
I am very concerned.	106.41	86.27	0.025
I am unsure how to evaluate the situation.	105.86	87.78	0.044
I believe the situation must be taken seriously.	104.86	90.48	0.108

**Table 3:** Differences between parents counseled before and during the pandemic with regard to different modifiers of counseling success (* Mann Whitney-U Test).

Modifiers of Counseling Success			
	Before COVID-19	During COVID-19	
*n* = 222	166	56	
	Mean rank		*p* *
Interruptions during counseling.	107.97	121.96	0.116
*n* = 222	167	55	
	Mean rank		*p* *
I would have preferred a separate counseling room.	111.09	112.74	0.863
*n* = 219	166	53	
	Mean rank		*p* *
Little time was lost after suspecting CHD in the fetus, making the diagnosis and counseling by a specialist.	109.99	110.00	0.999
*n* = 190	142	48	
	Mean rank		*p* *
I would have wished more detailed written explanatory information or links to useful online data.	100.32	81.23	0.034

**Table 4:** Mean values for parental counseling success—overall (a) and in the five analytical dimensions (b)—if complex fetal CHD was diagnosed: before and during the COVID-19 pandemic (1 = successful to 3 = unsuccessful).

	Before COVID-19	During COVID-19
	Mean Values	Mean Values
(a) Overall counseling success	1.58	1.70
(b) Dimensions:		
Transfer of medical knowledge	1.55	1.50
Trust in medical staff	1.33	1.39
Transparency regarding the treatment process	1.38	1.35
Coping resources	1.60	1.73
Perceived situational control	1.75	1.62

**Table 5:** Counseling success (COVID-subgroup only) in parents with another native language than the language counseling was conducted in (counseling via interpreters not included).

	Counseling Success		
	Successful	Satisfying	Unsuccessful
(a) Overall counseling success	57.1%	42.9%	-
(b) Dimensions:			
Transfer of medical knowledge	100%	-	-
Trust in medical staff	71.4%	28.6%	-
Transparency regarding the treatment process	100%	-	-
Coping resources	62.5%	37.5%	-
Perceived situational control	75.0%	12.5%	12.5%

## Supplementary Materials

The following are available online at https://www.mdpi.com/article/10.3390/jcm10153423/s1. Table S1: Overview of fetal cardiac diagnoses and associated chromosomal or extracardiac anomalies; Figure S1: Study flow-chart; Figure S2: Design of the questionnaire.
