# Supplementary material for: Fetal Cardiac Services during the COVID-19 Pandemic: How Does It Affect Parental Counseling?
Source: J Clin Med. 2021 Jul 31;10(15):3423. doi: 10.3390/jcm10153423 (PMC8347932; doi:10.3390/jcm10153423)
Supplement: Supplementary file 1 [file jcm-10-03423-s001.zip › Table S1.pdf]

Table S1. Overview of fetal cardiac diagnoses and associated chromosomal or extracardiac anomalies.

Fetal cardiac diagnosis	Genetic or extracardiac findings
ASD II	Trisomy 21
Aortic stenosis, aortoc arch hypoplasie, perimembranous VSD	
Aortic stenosis, coarctation	
ASD, LSVC	Trisomy 21
AVSD	Trisomy 21
AVSD	
AVSD	Trisomy 21
AVSD	
AVSD	
AVSD	Trisomy 21
AVSD	
AVSD	Trisomy 21
AVSD	
AVSD	Trisomy 21
AVSD	Trisomy 21
AVSD, ARSA	Trisomy 21
AVSD, hypoplastic aortic arch, coarctation	
AVSD, TOF	Trisomy 21
Borderline left ventricle, hypoplastic aortic arch, coarctation, LSVC	
ccTGA	
ccTGA	
Coarctation	
Coarctation	
Coarctation	
Coarctation	
Coarctation	Turner syndrome
Coarctation	
Coarctation, ARSA	
Complex TGA	
Complex TGA	
Complex TGA	
Complex TGA	
Complex TGA	
Complex TGA	
Complex TGA	
Complex TGA	
Complex TGA	
Complex TGA	
Critical aortic stenosis	
Critical aortic stenosis, severe mitral regurgitation, coarctation	
Critical pulmonary stenosis, severe tricuspid regurgitation	

DILV, MGA, aortic arch hypoplasia	
DILV, MGA, aortic arch hypoplasia, bilateral SVC	
DORV	
DORV	
DORV	
DORV	
DORV, aortic arch hypoplasia, coarctation	
DORV, MGA, right aortic arch, hypoplastic aortic arch, coarctation	
DORV, subpulmonary stenosis	
DORV, TGA	
DORV, TGA, PA	
DORV, TGA, subpulmonary stenosis	
DORV, TOF type, right aortic arch, MAPCA	
Ebstein´s anomaly of the tricuspid valve	
Ebstein´s anomaly of the tricuspid valve	
Heterotaxy syndrome, AVSD, absent right AV connection, pulmonary stenosis, MGA, bilateral SVCs	Situs inversus abdominalis, asplenia
Heterotaxy syndrome, AVSD, pulmonary stenosis, MGA, right aortic arch	
Heterotaxy syndrome, dextrocardia, DORV, pulmonary stenosis, MGA	
Heterotaxy syndrome, HLHS, TAPVR, azygos continuation	
HLHS	
HLHS	
HLHS	
HLHS	
HLHS	
HLHS	
HLHS	
HLHS	
HLHS, DORV	
HLHS, VSD	
Hydrops, tricuspid regurgitation	
Hypoplastic aortic arch	
Hypoplastic aortic arch	
Hypoplastic aortic arch, borderline LV	
Hypoplastic aortic arch, coarctation	
Hypoplastic aortic arch, coarctation	
Hypoplastic aortic arch, perimembranous VSD	
Hypoplastic aortic arch, VSD	*MCAD
Hypoplastic aortic arch, VSD muscular, PAPVR	
IAA, borderline left ventricle	DiGeorge-syndrome
IAA, VSD	
LAI, dextrocardia, hypoplastic right ventricle, tricuspid atresia, pulmonary stenosis, VSD, MGA	
LSVC	
Non-compaction cardiomyopathy	

PA, IVS	
PA, IVS	
PA, IVS, bipartite right ventricle, right ventricular hypertrophy	
PA, IVS, sinusoids	
PA, VSD	
PA, VSD	
PA, VSD	
PA, VSD	
PA, VSD, MAPCAs	
PA, VSD, TGA	
Pulmonary stenosis	
Right aortic arch	
Severe aortic stenosis	
Severe Ebstein´s anomaly of the tricuspid valve	
Severe pulmonary stenosis, severe tricuspid regurgitation	
Suspicion for coarctation, ventricular disproportion	
Suspicion for coarctation, ventricular disproportion	
TGA	
TGA (simple)	
TGA (simple)	
TGA (simple)	
TGA (simple)	
TGA (simple)	
TGA (simple)	
TGA (simple)	
TOF	Trisomy 21
TOF	
TOF	
TOF	
TOF	
TOF	
TOF	
TOF, right aortic arch	DiGeorge-syndrome
Tricuspid atresia	
Tricuspid atresia	
Tricuspid atresia Ib	
Tricuspid valve dysplasia, moderate regurgitation, mild pulmonary stenosis, LSVC	Trisomy 21
Tricuspid valve dysplasia, prenatal duct closure	
VSD	renal agenesis
VSD	
VSD	
VSD	
VSD	
VSD	*Reciprocal translocation chromosome 1 and 7; deletions: 1q43 and 7p15.3 - p21.1

VSD	*Cystic fibrosis
VSD inlet	
VSD muscular	
VSD perimembranous	
VSD, hypoplastic aortic arch, coarctation, LSVC	

*postnatal diagnosis

Abbreviations (in alphabetical order):

ARSA - aberrant right subclavian artery
AV - atrioventricular
ASD - atrial septal defect
AVSD - atrioventricular septal defect
CC - congenitally corrected
CHD - congenital heart disease
DORV - double outlet right ventricle
HLHS - hypoplastic left heart syndrome
IAA - interrupted aortic arch
IVS - intact ventricular septum
LAI - left atrial isomerism
LSVC - left superior vena cava
MAPCAs - main aortopulmonary collateral arteries
MCAD - medium-chain acyl-CoA dehydrogenase deficiency
MGA - malposition of the great arteries
PA - pulmonary atresia
PAPVR/TAPVR - partial or total anomalous pulmonary venous return
TGA - transposition of the great arteries
TOF - tetralogy of Fallot
VSD - ventricular septal defect
