# Supplementary figures and images for: Fetal Cardiac Services during the COVID-19 Pandemic: How Does It Affect Parental Counseling?
Source: J Clin Med. 2021 Jul 31;10(15):3423. doi: 10.3390/jcm10153423 (PMC8347932; doi:10.3390/jcm10153423)

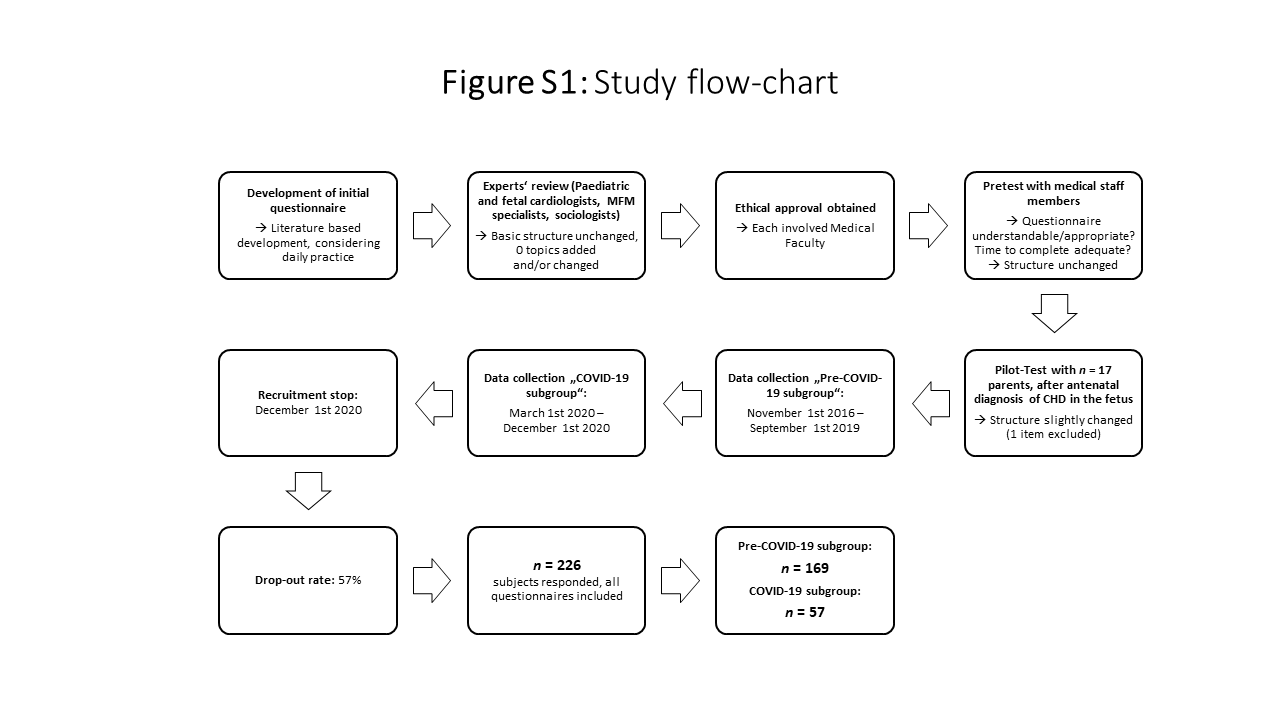

Supplement: Supplementary file 1 [file jcm-10-03423-s001.zip › Figure S1.tif]

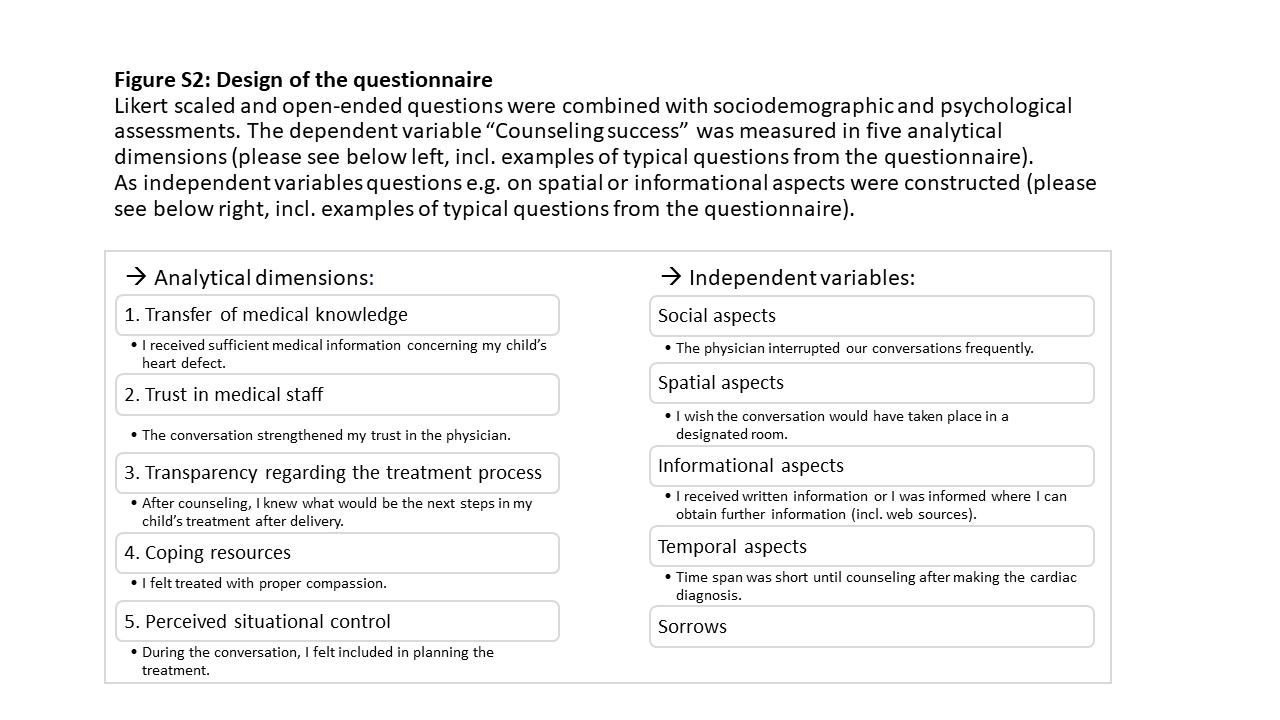

Supplement: Supplementary file 1 [file jcm-10-03423-s001.zip › Figure S2.tif]
